# Service delivery point and individual characteristics associated with the adoption of modern contraceptive: A multi-country longitudinal analysis

**DOI:** 10.1371/journal.pone.0254775

**Published:** 2021-08-17

**Authors:** Philip Anglewicz, Carolina Cardona, Titilope Akinlose, Peter Gichangi, Funmilola OlaOlorun, Elizabeth Omoluabi, Mary Thiongo, Pierre Akilimali, Amy Tsui, Patrick Kayembe, The PMA Agile Principal Investigators Group

**Affiliations:** 1 Department of Population, Family, and Reproductive Health, Johns Hopkins Bloomberg School of Public Health, Baltimore, Maryland, United States of America; 2 Research Triangle Institute International, Health Care Financing and Payment, Research Triangle Park, North Carolina, United States of America; 3 International Centre for Reproductive Health Kenya, Nairobi, Kenya; 4 University of Ibadan, Ibadan, Nigeria; 5 Centre for Research, Evaluation Resources and Development, Ife, Nigeria; 6 University of Kinshasa School of Public Health, Kinshasa, Democratic Republic of Congo; FHI360, UNITED STATES

## Abstract

**Background:**

Women who start using contraception (“adopters”) are a key population for family planning goals, but little is known about characteristics that predict the adoption of contraception as opposed to current use. We used prospective data from women and facilities for five countries, (Democratic Republic of Congo, India, Kenya, Nigeria, and Burkina Faso) and identified baseline characteristics that predicted adoption of modern contraception in the short term.

**Methods:**

We used data from the Performance Monitoring for Action (PMA) Agile Project. PMA Agile administered service delivery point (SDP) client exit interview (CEI) surveys in urban sites of these five countries. Female clients responding to the CEI were asked for phone numbers that were used for a phone follow-up survey approximately four months later. For our analysis, we used data from the SDP and CEI baseline surveys, and the phone follow up to compare women who start using contraception during this period with those who remain non-users. We used characteristics of the facility and the woman at baseline to predict her contraception adoption in the future.

**Results:**

Discussing FP with a partner at baseline was associated with greater odds of adoption in DRC (OR 2.34; 95% CI 0.97–5.66), India (OR 2.27; 95% CI 1.05–4.93), and Kenya (OR 1.65; 95% CI 1.16–2.35). Women who discussed family planning with any staff member at the health facility had 1.72 greater odds (95% CI 1.13–2.67) of becoming an adopter in Nigeria. The odds of adoption were lower in Nigerian facilities that had a stockout (OR 0.66 95% CI 0.44–1.00) at baseline. Other characteristics associated with contraception adoption across settings were education, age, wealth, parity, and marital status.

**Conclusions:**

Characteristics of both the woman and the health facility were associated with adoption of modern contraception in the future. Some characteristics, like discussing family planning with a spouse, education, and parity, were associated with contraceptive adoption across settings. Other characteristics that predict contraceptive use, such as health facility measures, varied across countries.

## Background

The number of additional users of modern contraception, a FP2020 Core Indicator, is calculated as the difference between the number of modern contraceptive users in a given year and the number of users at baseline, in 2012 (both of which are aggregated across the world’s 69 poorest countries) [1]. A component of the number of additional users is women who start using contraception, or “adopters” [2]. These adopters are therefore a key population for family planning goals.

The term “adoption” implies change, and measuring individual-level change in contraceptive use, and the factors that influence its uptake, involves several challenges [2]. The majority of individual-level analyses of modern contraceptive use in low- and middle-income settings use cross-sectional data that are often unable to accurately measure changes in contraceptive use over time. Much of the existing research on contraceptive adoption, discontinuation, and method switching uses the five year “contraceptive calendar” collected by Demographic and Health Surveys (DHS) [3–5]. While the contraceptive calendar is a valuable source of information, a disadvantage is that characteristics of women are only measured at the time of the survey instead of at the time of the adoption, discontinuation, or switching that may have taken place up to five years before the time of interview. This is important because some characteristics, such as wealth, urban residence, and fertility preferences may both affect and be impacted by contraceptive use [6–9]. As a result, a cross-sectional analysis of determinants with contraceptive calendar data does not permit one to distinguish between characteristics that facilitate use and those that change as a result of using contraception. Another challenge is that the calendar method can suffer from recall bias related to the use, timing, and type of contraception [10, 11]. As a result, relatively little is known about the characteristics that *predict* contraceptive adoption.

Another common limitation of research on adoption of contraceptive use is the lack of family planning service delivery characteristics. A woman’s decision to use contraception may be influenced by characteristics of the health system in her setting such as method availability, distance to the facility, facility type, and quality of care [12–17]. However, research on health facility characteristics typically cannot be merged with women’s longitudinal data, and measures factors associated with current contraceptive use instead of factors influencing at the time of adoption [8].

A prospective approach that measures contraceptive use over time (and associated characteristics) could address many of these challenges. In this case, because contraceptive use is measured at the time of the survey and not retrospectively, this approach may yield more accurate measures of contraceptive use and change than the contraceptive calendar. The need for such prospective longitudinal data to adequately measure adoption has been noted in previous research [2, 10]. However, few studies have used a prospective approach of following women over time to measure contraceptive change [18–21], and family planning service facility characteristics are often not included in research on contraceptive adoption.

In this research, we used data from five countries (the Democratic Republic of Congo, India, Kenya, Nigeria, and Burkina Faso) to identify baseline characteristics that predicted female clients’ modern contraception adoption in the future, focusing not only baseline characteristics of the woman, but also health facility characteristics such as service quality, availability of contraceptive methods, and cost of methods.

## Methods

### Data

Data for this study come from the Performance Monitoring for Action (PMA) Agile Project. PMA Agile was a continuous data monitoring and evaluation system that collected data every four to six months on the overall health service delivery environment. PMA Agile operated in urban areas of six countries, Burkina Faso, the Democratic Republic of Congo, India, Kenya, Niger, and Nigeria. PMA Agile had multiple sites in four countries: Lagos, Kano, and Ogun in Nigeria; Uasin Gishu, Migori, and Kiricho in Kenya; Indore, Firozabad, and Puri in India; Ouagadougou, and Koudougou in Burkina Faso. There was one PMA Agile site in each of the two remaining countries, Kinshasa, DRC; and Niamey, Niger. Data were collected in urban health facilities; the surveys were conducted at low cost with rapid turnaround. More information about PMA Agile can be found at the project’s website: www.pmadata.org/technical-areas/pma-agile, and in the PMA Agile Cohort Profile [22].

PMA Agile used a similar sampling approach in each country and site. PMA Agile started with a full list of public and private family planning service delivery points for each urban area, and then randomly selected 220 facilities in each site, with equal numbers public and private. The sample size accounted for 10% expected non-participation among selected facilities. PMA Agile then conducted both a service delivery point (SDP) and client exit interview (CEI) at each selected facility. The former was administered to a representative who was knowledgeable about the family planning services at the facility, and measured topics such as the availability of contraceptive methods and the cost of each method.

The CEI was administered to both male (aged 18–59) and female (18–49) facility clients who visited one of the facilities in the PMA Agile sample. The CEI was administered to approximately 10 clients per SDP, which yielded a sample size of 1,500–2,000 per PMA Agile site. CEI participants were selected systematically using a sampling interval that was calculated from the daily client flow reported from the SDP survey. The CEI survey instrument included questions on sociodemographic characteristics, contraceptive use, service quality, and family planning product recognition. A mobile airtime card with a value of about one USD was provided to each respondent completing the interview.

At the end of the CEI, female clients were asked if they would be willing to be followed up by telephone after four months and if so, to provide up to two telephone numbers. The same interviewer at baseline typically conducted the follow-up interview. Mobile phone airtime of one USD, transmitted electronically, was again provided to the followed-up female client. In the CEI follow-up survey, women were asked about continued contraceptive use, method switching, and satisfaction with services received.

We used CEI baseline and follow up data from five of the six countries, omitting Niger due to a small sample size of adopters. The CEI follow up was administered to women, so men are not included in our analysis. In all five countries, the baseline CEI and SDP surveys were conducted in 2018. In Burkina Faso, baseline data collection occurred from August through October; in DRC from May through June; In India from July through October; and in Kenya and Nigeria from March through August. The phone follow-up CEIs were conducted between four to six months later starting in September 2018 through April 2019 in all countries.

The PMA Agile study and data collection protocols were reviewed and approved by the Johns Hopkins Bloomberg School of Public Health Institutional Review Board and the in-country counterpart review board: Kenyatta National Hospital-University of Nairobi Ethics Research Committee (KNH-UoN ERC P470/08/2017); National Health Research Ethics Committee of Nigeria (NHREC/01/01/2007-19/09/2019); MOH-Burkina Comité d’Ethique pour la Recherche en Santé (MOH 2018-02-027); University of Kinshasa School of Public Health Institutional Review Board (ESP/CE/070/2017); Indian Institute for Health Management Research Ethical Review Board (19/12/2017-15/01-2018); MOH- Niger Comité National d’Ethique pour la Recherche en Santé (027/2020/CNERS). In accordance with country specific approved consent procedures, participants provided informed verbal consent in DRC, Kenya, India, and Nigeria (consent was recorded in a checkbox by the interviewer into the smartphone); and participants provided informed written consent in Burkina Faso. The data used in this analysis were completely anonymised, deidentified, and aggregated before access and analysis.

### Measures

Our measures are consistent with the standard approaches. We defined women who adopted modern contraception as “someone who starts using family planning who was not currently using modern contraception at the time of her visit but may have used modern contraception in the past” [2]. This definition included both women who used modern contraception in the past but discontinued use before adopting again, as well as first-time users of modern contraception. Following WHO standards, we defined the following contraceptive methods as modern: oral pills, intrauterine devices, injectables, male and female sterilization, implants, condom, lactational amenorrhea method, vaginal barrier methods, emergency contraception, and cycle beads.

To measure adoption, we used PMA Agile data from women interviewed in both the in-person baseline CEI and the telephone CEI follow-up, and limited our analysis to women who were not using any contraceptive method at baseline. Modern contraceptive method use at baseline was determined through two measures from the CEI survey. Clients that were attending an SDP seeking services besides family planning were asked about their current contraceptive use status. Among clients who were attending an SDP seeking family planning services, use status was assigned from the contraceptive method either prescribed or given. To capture contraceptive use at the follow-up survey, women were directly asked about their current contracepting status. Adopters were defined as female clients who were not using modern contraception at baseline and reported using modern contraception at the time of the follow-up survey. We compared these adopters with *continued non-users of contraception*, who were female clients not using a contraceptive method at baseline or follow up. Women using traditional contraceptive methods were included among non-users of modern contraception. Women using modern methods at baseline were excluded from this analysis.

To identify the predictors of contraceptive adoption, we used CEI and SDP baseline characteristics. Our selection of specific characteristics was guided by the literature on this topic. We started with sociodemographic characteristics, such as age (separated into three categories, 18–24, 25–34, 35–49), parity (none, one, two, three or more), education (none/primary, secondary, higher), and marital status (currently married, not currently married). We used the Cantril ladder to measure household wealth, in which female clients ranked their household wellbeing on a 10-step staircase where the first step represents the poorest and the 10th step represents the richest (we separate into 1–3, 4, 5, 6–10) [23]. Research has shown that spousal communication is often associated with family planning use [24], so we included a measure of whether the woman discussed family planning use with their partner in the past six months. We included exposure to family planning programs, measured by seeing advertisement for FP on radio or television in the past three months, and being visited by a community health worker and discussing FP with a health care provider in the past 12 months. Media exposure to family planning was not collected in India. We distinguished between several reasons for the visit to the facility (family planning/maternal health, child health, general health/other). Reasonable access to a health facility is also associated with contraceptive use [17], so we included a measure of distance to the facility (less than 1 kilometer, between 1 and the median distance, and above the median distance).

Our measures of facility characteristics focus on general service quality from the visit that took place on the day of the baseline interview. Women were asked about common problems clients face at health facilities and whether they experienced any of these problems at their visit. They were asked whether each of these items were a problem on that day, with response options ranging from 0 to 2, 0 being not a problem and 2 a major problem. The problems listed were time waited to see a provider, cleanliness of the facility, and cost of services or treatment. We also included an indicator of whether staff at the health facility discussed family planning during the visit.

We also included measures of facility characteristics from the SDP survey. Specifically we measured the facility type (hospital, health center, pharmacy, other), as service quality and cost of contraceptives often vary by type [25]. Our categorization of facility types represented the four broadest categories that had comparable types across settings. Alternative categorizations did not change the substantive interpretation of our results. Distributions of SDP types prior to our recoding appear in S1 Table. The cost of contraception has been found to influence contraceptive behavior [26], so we included a dichotomous variable indicating if the facility where the client was interviewed at baseline charged a fee for providing family planning methods. Finally, we included contraceptive availability, measured as a binary variable that equal to one if the facility had any method out-of-stock or if they are not offered, and zero if they have all methods in stock, either short-acting or long-acting. These characteristics have all been identified as important potential influences on contraceptive use [12–15, 27].

### Statistical approach

We conducted our analysis in four steps. First, we presented sample numbers and response rates for each PMA Agile country. Next, we showed the percentage of women who were adopters and non-users in each country. Third, we tabulated percentages of all characteristics that influenced adoption, and performed bivariate chi-squared tests of differences in these characteristics between adopters and continuing non-users (separately for each country). Finally, we constructed a logistic regression model with site fixed effects to identify baseline characteristics that predicted modern contraception adoption in the future. Our outcome of interest is the proportion of female clients defined as adopters, which is characterized as a function of service delivery point and individual characteristics resulting in the following function:

$${\text{Adopter}}_{\text{i},\text{t}+1}=\alpha_{\text{i}}+\beta_{1}\;{\text{SDP}}_{\text{i,t}}+\beta_{2}\;{\text{SES}}_{\text{i,t}}+\beta_{3}\;{\text{FPComm}}_{\text{i,t}}+\beta_{4}\;{\text{FPExposure}}_{\text{i,t}}+\beta_{5}\;{\text{RF}}_{\text{i,t}}+\delta_{\text{s}}+\varepsilon_{\text{it}}$$

In this equation “Adopter” represents the proportion of female clients who adopted a modern contraceptive method in period *t+1* given they were not using contraception in period *t* (*t* = baseline) for each site indexed by “*i"* (*i* = 1 in DRC, *i* = 2 in Burkina Faso and *i* = 3 in Kenya, Nigeria, and India). SDP_i,t_ is a vector that represents service delivery point characteristics. SES_i,t_ is a vector that represents the client’s sociodemographic characteristics. FPComm_i,t_ represents spousal communication about family planning in the last six months. FPExposure_i,t_ represents exposure to family planning information. RF_i,t_ represents the relationship between the client and the facility by capturing the visit reason and distance to the facility. All service delivery point and individual characteristics were measured at time *t*. Finally, δ_s_ represents site fixed effects; and ε_it_ is the error term. The analysis was performed separately by country. For these regression models, we show odds ratios (OR) and 95% confidence intervals (95% CIs).

As a robustness check we specified two additional models following a similar approach but restricting the covariates of the model. The first model only included SDP characteristics, while the second model only included socioeconomic factors, exposure to family planning, spousal communication, and the relationship between the client and the facility. We also tested for multicollinearity through variance inflation factors (VIFs) and did not include any measures that exceeded a value of 7.0 for VIF.

## Results

Table 1 shows sample numbers for female clients interviewed in baseline and follow-up. The majority of women eligible for the CEI follow up were successfully interviewed in PMA Agile countries. The percentage of women consenting to follow up was over 80% in all countries except India (at 70%). The percentage of women with a successful follow up interview ranged from 59% in India to 91% in Kenya. We limit our sample to data from non-contracepting women of reproductive ages with complete information at both baseline and follow-up, resulting in sample sizes of 315 women living in DRC, 349 in India, 1,189 in Kenya, 1,493 in Nigeria, and 572 in Burkina Faso.

**Table 1:** Female client interviews at baseline and follow-Up in Burkina Faso, DRC, India, Kenya and Nigeria, PMA Agile 2017–2020.

	Dem. Rep. of Congo	India	Kenya	Nigeria	Burkina Faso
Completed baseline interviews for women (N)	1,226	1,596	4,431	3,752	1,512
Women consenting to follow-up (%)	83%	70%	98%	85%	94%
Women with completed follow-up interviews (%)	75%	59%	91%	73%	84%

**Note**: Not all women agreed to the follow-up interview, and among those who did, not all were successfully re-interviewed. More information about the PMA Agile interview procedures and response rates can be found in Tsui et al. 2020.

**Fig 1:**
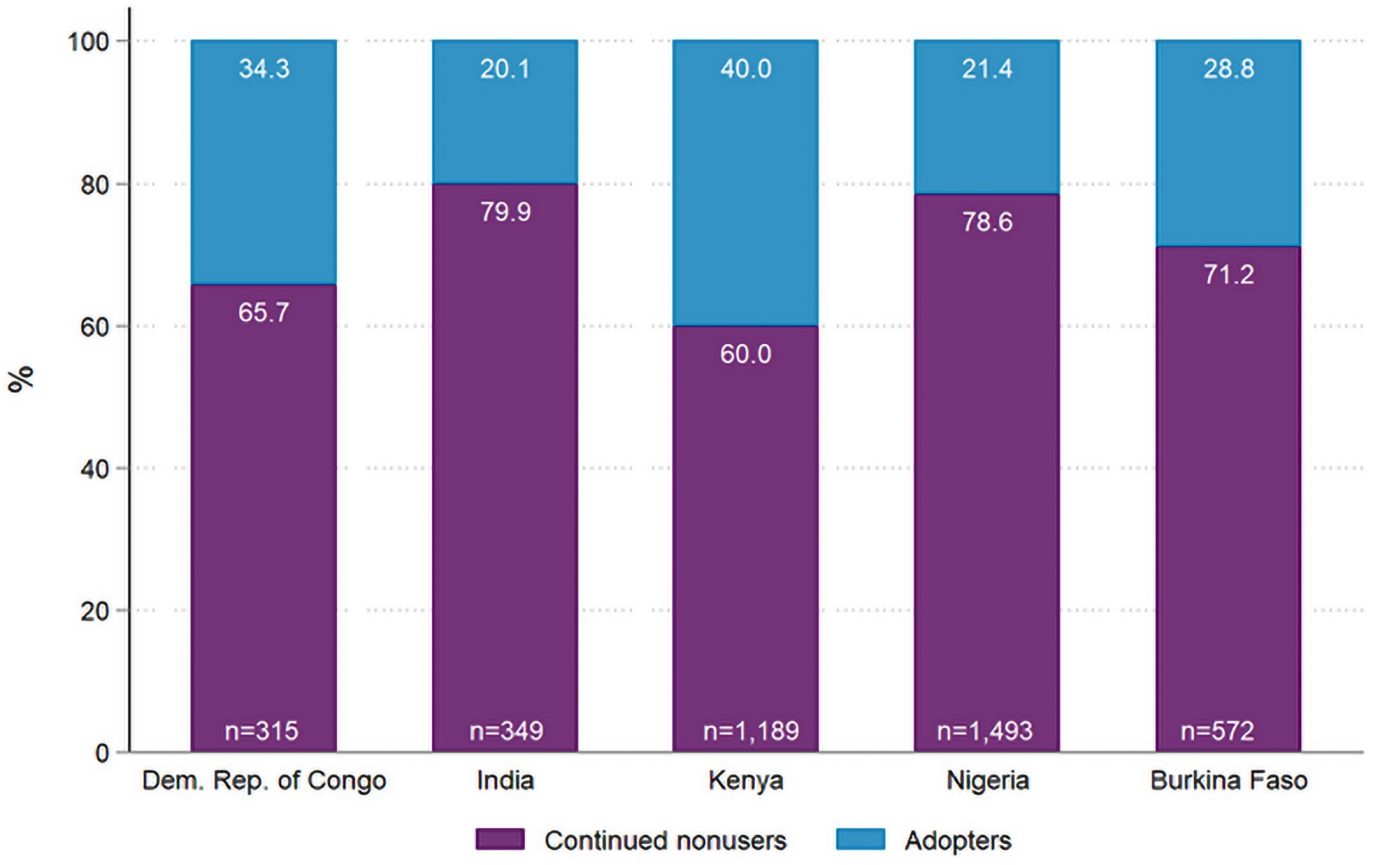
Percentages of continuing non-users and adopters in the Democratic Republic of Congo, India, Kenya, Nigeria, and Burkina Faso, PMA Agile 2017–2020.

Fig 1 shows the percentages of our measure of primary interest, continuing non-users and adopters of modern contraception. Variation across countries in percentages of women adopting contraception is evident in Fig 1, as the percentage of adopters ranged from 20% in India to 40% in Kenya. We also show the distribution of methods used among those adopting between baseline and follow-up in S1 Fig. The method mix among adopters was different across countries, the majority of clients adopted the pill in India, while in Kenya and Nigeria they adopted the injectable, in DRC the male condom, and in Burkina Faso the IUD. The difference in sample sizes between Table 1 and Fig 1 is because the latter (and our subsequent analysis) does not include women who continued to use contraception in both baseline and follow-up or women who discontinued using a contraceptive method between baseline and follow up.

Individual and service delivery point characteristics are shown in Table 2, separately for continuing non-users (NU) and adopters (A), and with chi-squared tests of differences between these groups. Among SDP characteristics, continuing non-users and adopters had significant differences in managing authority, with higher percentages of adopters visiting public facilities at baseline in India and Nigeria; and the cleanliness of the facility, but only in DRC, where cleanliness was a problem for 27.4% of adopters and 17.1% of continuing non-users. As for family planning method fees, we found significant differences in DRC, Nigeria, and Burkina Faso. In Nigeria, the majority of continuing non-users (53.7%) and adopters (65.6%) attended a facility where contraceptives were offered free of charge. Contraceptive stocks were significantly different between continuing non-users and adopters only in Burkina Faso. The percentage of continuing non-users attending a facility that does not offer modern contraception or has them out-of-stock is larger than the percentage of adopters attending such facilities, 52.9% and 42.2%, respectively. The distribution of clients across facility types varies between countries. Only Nigeria and Burkina Faso had a similar pattern, in which most nonusers and adopters attended a health center, 44.0% and 53.4%, and 53.3% and 60.6%, respectively. In India, a pharmacy—41.6% of nonusers and 34.3% of adopters—and in DRC a health center—58.3% of nonusers and 54.3% of adopters—were the most common facility type in the sample. We also found that women who discussed FP with staff at the facility were significantly more likely to adopt in India, Kenya, and Nigeria.

**Table 2:** Percent distribution of individual and service delivery point characteristics for continuing non-users and adopters at baseline in the Democratic Republic of Congo, India, Kenya, Nigeria, and Burkina Faso.

	Dem. Rep. of Congo			India			Kenya			Nigeria			Burkina Faso		
	[NU]	[A]	Chi^2^ test	[NU]	[A]	Chi^2^ test	[NU]	[A]	Chi^2^ test	[NU]	[A]	Chi^2^ test	[NU]	[A]	Chi^2^ test
	%	%	p-value	%	%	p-value	%	%	p-value	%	%	p-value	%	%	p-value
**Managing authority**															
Private	37.9	43.4	0.34	**73.8**	**62.9**	**0.07**	46.6	47.3	0.81	**44.0**	**30.9**	**0.00**	41.4	34.4	0.13
Public	62.1	56.6		**26.2**	**37.1**		53.4	52.7		**56.0**	**69.1**		58.6	65.6	
**Time you waited to see a provider**															
Not a Problem	84.5	80.2	0.34	86.0	84.3	0.72	90.4	92.6	0.19	92.4	90.9	0.39	85.0	86.1	0.75
Problem	15.5	19.8		14.0	15.7		9.6	7.4		7.6	9.1		15.0	13.9	
**Cleanliness of the facility**															
Not a Problem	**82.9**	**72.6**	**0.03**	94.6	95.7	0.70	96.1	94.7	0.28	97.3	96.6	0.50	92.9	93.3	0.84
Problem	**17.1**	**27.4**		5.4	4.3		3.9	5.3		2.7	3.4		7.1	6.7	
**Cost of services or treatment**															
Not a Problem	80.2	76.2	0.42	89.2	82.9	0.15	93.4	93.5	0.95	95.4	95.9	0.67	94.3	95.8	0.49
Problem	19.8	23.8		10.8	17.1		6.6	6.5		4.6	4.1		5.7	4.2	
**Facility type**															
Hospital	19.0	17.6	0.73	25.1	24.3	0.57	8.3	7.8	0.91	**26.3**	**13.1**	**0.00**	**9.8**	**9.1**	**0.06**
Health Center	58.5	55.9		16.8	22.9		10.9	10.1		**44.0**	**53.4**		**53.6**	**59.4**	
Other^a^				16.5	18.6		58.1	60.1		**16.1**	**20.3**		**26.0**	**16.4**	
Pharmacy	22.4	26.5		41.6	34.3		22.7	22.1		**13.6**	**13.1**		**10.6**	**15.2**	
**Fee for FP methods**															
No	28.6	17	**0.02**	35.5	41.4	0.36	64.1	63.9	0.94	53.7	65.6	**0.00**	38.8	29.9	0.05
Yes	71.4	83		64.5	58.6		35.9	36.1		46.3	34.4		61.2	70.1	
**Contraceptive stocks**															
In-stock	39.3	44.3	0.39	33.7	37.1	0.59	65.9	65.5	0.89	63.0	65.9	0.33	47.1	57.8	**0.03**
Not offered/Out-of-stock	60.7	55.7		66.3	62.9		34.1	34.5		37.0	34.1		52.9	42.2	
**Staff at health facility discuss FP**															
No	96.1	92.4	0.16	**97.1**	**91.4**	**0.03**	**95.5**	**92.9**	**0.05**	**87.5**	**81.3**	**0.00**	94.1	91.5	0.26
Yes	3.9	7.6		**2.9**	**8.6**		**4.5**	**7.1**		**12.5**	**18.8**		5.9	8.5	
**Visit reason**															
FP & Maternal Health	26.7	20.8	0.09	**28.0**	**18.6**	**0.03**	16.4	15.8	0.85	**26.8**	**16.9**	**0.00**	**24.8**	**27.3**	**0.00**
Child Health	29.1	41.5		**18.6**	**32.9**		30.4	29.4		**36.8**	**45.6**		**31.2**	**46.1**	
General Health & Other	44.2	37.7		**53.4**	**48.6**		53.2	54.8		**36.4**	**37.5**		**44.0**	**26.7**	
**Distance to facility**															
Less than 1 km	49.3	49.5	0.96	17.3	14.3	0.40	30.2	29.6	0.90	43.2	42.5	0.81	15.2	17.8	0.44
1—median km ^b^				41.0	50.0		22.9	22.1		20.0	21.8		39.1	42.3	
Median+ km	50.7	50.5		41.7	35.7		47.0	48.3		36.8	35.7		45.7	39.9	
**Age group**															
18–24	30.1	32.1	0.92	33.0	30.0	0.20	**43.8**	**34.7**	**0.00**	22.3	23.1	0.96	**35.6**	**38.8**	**0.02**
25–34	41.7	41.5		47.0	40.0		**33.1**	**42.4**		50.2	49.7		**41.0**	**48.5**	
35–49	28.2	26.4		20.1	30.0		**23.1**	**22.9**		27.5	27.2		**23.3**	**12.7**	
**Parity group**															
None	27.2	22.6	0.83	**39.4**	**11.4**	**0.00**	**27.6**	**19.7**	**0.02**	**27.7**	**14.7**	**0.00**	**33.7**	**22.4**	**0.00**
One	23.8	25.5		**29.0**	**28.6**		**21.6**	**22.9**		**21.1**	**21.3**		**19.4**	**32.1**	
Two	17.5	17.0		**19.4**	**32.9**		**17.1**	**20.2**		**19.6**	**24.7**		**17.9**	**18.2**	
Three +	31.6	34.9		**12.2**	**27.1**		**33.7**	**37.2**		**31.6**	**39.4**		**29.0**	**27.3**	
**Schooling**															
None/Primary	16.0	26.7	0.07	20.8	27.1	0.42	40.3	42.9	0.15	**9.4**	**11.7**	**0.01**	31.6	25.5	0.25
Secondary	57.3	52.4		58.1	50.0		35.6	37.8		**45.6**	**53.3**		38.3	44.8	
College / University	26.7	21.0		21.1	22.9		24.1	19.3		**45.0**	**34.9**		30.1	29.7	
**Household Wealth**															
Poorest 1–3	**30.9**	**48.6**	**0.00**	23.7	35.7	0.20	23.7	25.6	0.59	23.7	20.7	0.57	54.9	54.9	0.94
4	**22.5**	**22.9**		22.9	18.6		29.0	30.7		21.1	21.7		14.7	16.5	
5	**34.8**	**14.3**		26.5	25.7		32.7	29.0		27.9	31.2		21.2	19.5	
Richest 6–10	**11.8**	**14.3**		26.9	20.0		14.6	14.7		27.3	26.4		9.2	9.1	
**Marital status**															
In Union	63.6	54.7	0.13	**85.3**	**94.3**	**0.04**	59.2	63.7	0.12	**81.9**	**88.4**	**0.01**	75.4	81.2	0.14
Not in Union	36.4	45.3		**14.7**	**5.7**		40.8	36.3		**18.1**	**11.6**		24.6	18.8	
**Discussed FP w/Partner**															
No	68.0	70.8	0.61	**82.2**	**66.7**	**0.00**	**86.7**	**81.0**	**0.01**	80.3	75.9	0.09	**82.3**	**71.5**	**0.00**
Yes	32.0	29.2		**17.8**	**33.3**		**13.3**	**19.0**		19.7	24.1		**17.7**	**28.5**	
**Heard FP in radio or TV**															
No	67.0	59.4	0.19	^c^	^c^		59.3	62.2	0.32	73.4	78.1	0.08	73.7	72.1	0.70
Yes	33.0	40.6		N/A	N/A		40.7	37.8		26.6	21.9		26.3	27.9	
**Visited by CHW that talked about FP**															
No	81.0	76.4	0.35	88.3	81.8	0.16	90.6	88.8	0.32	92.0	90.3	0.35	93.9	92.7	0.61
Yes	19.0	23.6		11.7	18.2		9.4	11.2		8.0	9.7		6.1	7.3	
N	207	108		279	70		713	476		1,173	320		407	165	

**Note**: [NU] = Continued nonuser; [A] = Adopter; bold values indicate statistical significance at the p≤0.05 level;

^a^ = there were no “other” types of facilities in DRC;

^b^ = Median distance to facility in the Democratic Republic of Congo was less than 1 km. away from the SDP;

^c^ = media exposure to family planning was not collected in India.

We found no significant differences in the average age by user status in DRC, Kenya, and Nigeria; however, adopters in India were 1.9 years older than continuing non-users, while in Burkina Faso adopters were 1.6 years younger than continuing non-users. Differences across parity groups between continuing non-users and adopters were significant in India, Kenya, Nigeria, and Burkina Faso. Among adopters, there was a higher concentration of women at parity three or above, while it is lower for nulliparous women. The largest difference was registered in Nigeria where 39.4% of adopters had three or more children and 14.7% were nulliparous. There was no clear pattern across parity groups for continuing non-users. In both groups most female clients were in union, for adopters it ranged from 54.7% in DRC to 94.3% in India, while for continuing non-users it ranged from 63.6% in DRC to 85.3% in India. Even though the majority were in union, less than one-third of clients had discussed family planning with their partner in the last 6 months.

There were significant differences across clients’ main reason for visiting the health facility and with user status in DRC, India, Nigeria, and Burkina Faso. Among continuing non-users, the majority were seeking for general health and other services—ranging from 36.4% in Nigeria to 53.4% in India—while among adopters, outside of India, the majority were seeking child health services.

Table 3 presents the results of the logistic regression analysis with site fixed effects, in which we examined the association between SDP and individual characteristics and adopting modern contraception. Among SDP characteristics, we find that in Nigeria, the type of health facility, contraceptive stock-outs, and having discussed family planning with health care providers had a significant association with the odds of women becoming an adopter. Nigeria women who attended a health facility that either did not provide family planning methods or had them out-of-stock had lower odds of adopting contraception by a factor of 0.68 (95% CI 0.45–1.04). Among Nigerian women, the odds of becoming an adopter who went to a pharmacy were higher by a factor of 2.61 (95% CI 1.38–4.93) compared to women attending a hospital. We also found that Nigerian women who discussed family planning with any staff member at the health facility had 1.72 times greater odds (95% CI 1.12–2.66) of becoming an adopter than women whose provider did not discuss family planning; there are similarly higher odds of adoption in DRC, India, and Kenya, but results are not statistically significant. We also found that women in India who stated that the cost of services or treatment was a problem (compared to not being a problem) had greater odds of adoption (OR 3.56; 95% CI 1.14–11.13).

**Table 3:** Logistic regression model of service delivery point and individual characteristics associated with the probability that a client becomes an adopter over a continued nonuser in the Democratic Republic of Congo, India, Kenya, Nigeria, and Burkina Faso.

	Dem. Rep. of Congo	India	Kenya	Nigeria	Burkina Faso
	OR (95% CI)	OR (95% CI)	OR (95% CI)	OR (95% CI)	OR (95% CI)
**Managing authority**					
Private *(ref*.*)*					
Public	0.60 (0.17, 2.11)	5.94* (0.75, 46.92)	1.03 (0.70, 1.51)	1.18 (0.63, 2.21)	0.94 (0.54, 1.62)
**Time you waited to see a provider**					
Not a Problem *(ref*.*)*					
Problem	0.82 (0.27, 2.45)	0.77 (0.29, 2.05)	0.72 (0.46, 1.13)	1.47 (0.86, 2.51)	1.20 (0.64, 2.27)
**Cleanliness of the facility**					
Not a Problem *(ref*.*)*					
Problem	0.83 (0.31, 2.23)	0.31 (0.05, 1.88)	1.27 (0.71, 2.29)	1.21 (0.53, 2.79)	0.95 (0.44, 2.08)
**Cost of services or treatment**					
Not a Problem *(ref*.*)*					
Problem	1.96 (0.72, 5.33)	3.56** (1.14, 11.13)	0.89 (0.53, 1.48)	0.82 (0.38, 1.77)	0.61 (0.20, 1.83)
**Facility type**					
Hospital *(ref*.*)*					
Health Center	1.33 (0.41, 4.28)	0.73 (0.17, 3.14)	1.05 (0.59, 1.86)	1.06 (0.60, 1.89)	1.11 (0.51, 2.41)
Other		0.93 (0.31, 2.75)	1.09 (0.68, 1.74)	1.17 (0.62, 2.19)	0.87 (0.36, 2.13)
Pharmacy	0.43 (0.06, 3.13)	0.80 (0.26, 2.46)	0.83 (0.44, 1.57)	2.61*** (1.38, 4.93)	2.14 (0.66, 6.96)
**Fee for FP methods**					
No (*ref*.)					
Yes	1.50 (0.40, 5.54)	2.25 (0.44, 11.41)	1.19 (0.76, 1.87)	0.65 (0.38, 1.13)	0.85 (0.41, 1.78)
**Contraceptive stocks**					
In-stock (*ref*.)					
Not offered/Out-of-stock	2.49 (0.68, 9.19)	2.35* (0.88, 6.26)	1.10 (0.75, 1.61)	0.68* (0.45, 1.04)	0.75 (0.33, 1.71)
**Staff at health facility discuss FP**					
No *(ref*.*)*					
Yes	1.72 (0.25, 11.90)	3.35 (0.72, 15.59)	1.48 (0.87, 2.52)	1.72** (1.12, 2.66)	1.00 (0.44, 2.26)
**Visit reason**					
FP & Maternal Health *(ref*.*)*					
Child Health	3.18** (1.17, 8.63)	0.99 (0.34, 2.84)	0.95 (0.64, 1.41)	1.61** (1.03, 2.50)	1.07 (0.62, 1.85)
General Health & Other	1.29 (0.43, 3.83)	0.62 (0.23, 1.68)	1.24 (0.85, 1.81)	2.55*** (1.60, 4.08)	0.46** (0.22, 0.93)
**Distance to facility**					
Less than 1 km *(ref*.*)*					
1—median km		2.96** (1.03, 8.49)	0.91 (0.65, 1.28)	1.15 (0.77, 1.71)	0.77 (0.43, 1.40)
median+ km	1.20 (0.54, 2.66)	2.10 (0.70, 6.33)	1.12 (0.83, 1.50)	0.95 (0.67, 1.33)	0.70 (0.39, 1.27)
**Age group**					
18–24 *(ref*.*)*					
25–34	0.91 (0.37, 2.27)	0.33** (0.14, 0.80)	1.58*** (1.14, 2.18)	0.76 (0.51, 1.13)	0.74 (0.44, 1.24)
35–49	1.27 (0.37, 4.41)	0.74 (0.23, 2.35)	1.23 (0.79, 1.89)	0.54** (0.32, 0.91)	0.44** (0.20, 0.96)
**Parity group**					
None *(ref*.*)*					
One	1.19 (0.40, 3.55)	4.26** (1.38, 13.18)	1.41* (0.96, 2.09)	1.79** (1.01, 3.16)	2.07** (1.05, 4.09)
Two	0.90 (0.24, 3.30)	10.60*** (3.14, 35.85)	1.34 (0.85, 2.12)	2.22*** (1.22, 4.03)	1.43 (0.63, 3.25)
Three +	1.00 (0.27, 3.77)	14.50*** (3.39, 62.07)	1.15 (0.70, 1.89)	2.15** (1.17, 3.95)	1.96 (0.81, 4.73)
**Schooling**					
None / Primary *(ref*.*)*					
Secondary	2.65** (1.01, 6.99)	1.35 (0.58, 3.14)	1.15 (0.86, 1.55)	1.09 (0.64, 1.86)	1.68* (1.00, 2.84)
College / University	1.67 (0.48, 5.83)	3.85** (1.30, 11.37)	0.84 (0.56, 1.26)	0.83 (0.46, 1.48)	1.85* (0.92, 3.73)
**Household Wealth**					
Poorest 1–3 *(ref*.*)*					
4	0.54 (0.22, 1.34)	0.75 (0.30, 1.89)	0.95 (0.68, 1.32)	1.69** (1.07, 2.64)	1.06 (0.58, 1.94)
5	0.22*** (0.07, 0.68)	1.04 (0.43, 2.56)	0.85 (0.59, 1.21)	1.96*** (1.27, 3.02)	1.02 (0.58, 1.78)
Richest 6–10	1.22 (0.34, 4.41)	0.44 (0.15, 1.27)	0.99 (0.63, 1.54)	1.45 (0.92, 2.27)	1.37 (0.65, 2.92)
**Marital status**					
In Union *(ref*.*)*					
Not in Union	1.90 (0.74, 4.87)	0.67 (0.14, 3.11)	1.13 (0.83, 1.53)	0.59* (0.33, 1.04)	1.28 (0.62, 2.68)
**Discussed FP w/Partner**					
No *(ref*.*)*					
Yes	2.34* (0.97, 5.66)	2.27** (1.05, 4.93)	1.65*** (1.16, 2.35)	1.12 (0.79, 1.60)	1.45 (0.85, 2.46)
**Heard FP in radio or TV**					
No *(ref*.*)*					
Yes	0.63 (0.27, 1.49)		0.96 (0.74, 1.25)	1.12 (0.79, 1.59)	0.99 (0.62, 1.58)
**Visited by CHW that talked about FP**					
No *(ref*.*)*					
Yes	0.87 (0.36, 2.10)	1.16 (0.48, 2.79)	1.13 (0.76, 1.68)	1.41 (0.83, 2.37)	0.91 (0.41, 2.03)
Constant	0.08* (0.01, 1.07)	0.01*** (0.00, 0.09)	0.30*** (0.13, 0.70)	0.12*** (0.04, 0.34)	0.39 (0.09, 1.66)
**Observations**	275	325	1,181	1,204	514

**Note**:

*p<0.10;

**p<0.05;

***p<0.01;

odds ratios and 95% confidence interval in parenthesis; bold values indicate statistical significance at the p<0.05 level; a = there were only 10 other facilities in DRC, these observations were dropped in the multivariate analysis due to large standard errors; b = median distance to facility in the Democratic Republic of Congo was less than 1 km. away from the SDP; c = media exposure to family planning was not collected in India.

Regarding individual-level measures, many of the characteristics associated with adoption of a modern contraceptive varied across settings (Table 3). For example, women aged 25–34 had greater odds of adoption (compared to women aged 18 to 24) in Kenya, but lower odds in India. Women aged 35 to 49 also had lower odds of adoption than women aged 18 to 24 in Nigeria and Burkina Faso. There were similar results for household wealth, where women who reported the 5^th^ wealth level (out of 10) were less likely to adopt (compared to the lowest reported wealth) in DRC (OR 0.22; 95% CI 0.07–0.68), but were more likely to adopt in Nigeria (OR 1.95; 95% CI 1.27–3.02). There were also differences across settings in the relationship between adoption and visit reason: women visiting the facility for reasons related to child health had greater odds of adoption in Nigeria (OR 1.61; 95% CI 1.03–2.50) and women seeking general health services had lower odds in Burkina Faso (OR 0.46; 95% CI 0.22–0.93) (compared to FP and maternal health visits).

Some results were, however, consistent across countries in our analysis. Discussing FP with a partner was associated with greater odds of adoption in DRC (OR 2.34; 95% CI 0.97–5.66), India (OR 2.27; 95% CI 1.05–4.93), and Kenya (OR 1.65; 95% CI 1.16–2.35). In Burkina Faso, India, Kenya, and Nigeria, higher parity was associated with greater odds of adoption, compared to women with no children. There was a consistent relationship between education and adoption; where women with secondary education were more likely to adopt modern contraception compared to women with no education in DRC (OR 2.65 95% CI 1.01–6.99) and Burkina Faso (OR 1.68; 95% CI 1.00–2.84); and women with college/university education had higher odds of adopting contraceptive use than women with no/primary education in India (OR 3.85; 95% CIs 1.30–11.37) and Burkina Faso (OR 1.85; 95% CIs 0.92–3.73).

To address the robustness of these findings we specified two additional models, the first of which included only individual-level measures, and the second with only SDP characteristics (S2 and S3 Tables). In these models we identified the same significant factors with the same direction but with slightly different magnitude in some cases. Overall, these robustness tests do not impact the substantive interpretation of our main results.

## Discussion

In this research, we use prospective data from client exit interviews at service delivery points in urban areas of Burkina Faso, DRC, India, Kenya, and Nigeria to identify women who start using modern contraception over time, and compare these women to continuing non-users. We identify the baseline characteristics both of the woman and the service delivery point that may be associated with future adoption, and compare these characteristics across settings.

Many of our findings are compatible with previous research. Some facility characteristics appear to be influences on contraceptive use, as has been found elsewhere [12, 13, 17]. Women who were interviewed at pharmacies (in Nigeria) at baseline had greater odds of adopting modern contraception in the future than women interviewed at hospitals. The connection between these facility types and contraceptive use may reflect the better quality of care that is often provided at health clinics and pharmacies, compared to hospitals, which are sometimes characterized by lower quality counseling and greater inefficiency; as well as greater availability of certain methods [25]. Discussing FP with facility staff at baseline was associated with greater odds of adoption in Nigeria. This result is ambiguous, however, since it’s not clear whether women who were interested in FP initiated the discussion of FP or whether staff trained to discuss FP impacted contraceptive adoption- although we suspect that both may be the case, given that the percentage of clients that adopted contraception after attending the facility seeking family planning services ranged from 16% in Kenya to 27% in Burkina Faso. Finally, distance to the service delivery facility, which has influenced contraceptive use in some settings but not others [17, 28], is not associated with contraceptive adoption in any of our five countries. This may be due to the urban setting for our study, where facilities are many and transportation is relatively easy, and because our sample selects clients who were able to visit the facility.

Few of the facility service quality measures were significantly related to adoption. Among service quality measures, only women who reported that the cost of services (in India) were problems had greater odds of adopting contraception. This may be because if a woman does not intend to use contraception in the future, the cost of contraceptive products are not relevant (and therefore not problematic). We also tested other measures of quality, including a rating of the facility compared to others, if they were treated politely by staff, and overall satisfaction with the services, but none of these were statistically significant and were not included in the final models. In addition, many of the quality measures had too little variation to include in our models, as the vast majority of women reported positive experiences in their visit (described further below).

It is important to note that adopters may not have obtained their contraception at the facility where they were interviewed at baseline, and as a result, some of the baseline facility characteristics may differ from those of the facility that ultimately provided the contraception. However, we feel that the baseline facility characteristics may still impact adoption for two reasons. First, we expect that many women in fact returned to the same facility to obtain contraception. Clients are often loyal to one particular facility, particularly if the client believes that it provides better quality care than others [25]. Across these five PMA Agile settings, over 98% of clients in all five countries reported at baseline that they would return to the facility in the future, over 85% said the facility was better than other health care establishments, and over 95% of clients reported that they were treated politely by staff. Second, the experience at baseline facility may have a long-lasting impact on family planning-related behavior. For example, discussing FP with facility staff may impact contraceptive use even if the contraceptive is obtained at a different facility. In addition, clients may become more comfortable and confident with health services after a successful visit, and a negative experience at a health facility may deter clients from returning to any health facility [29].

In addition, like previous research [12, 30], we find that women with larger numbers of children are more likely to adopt modern contraception. Similarly, several studies have found that women with more children are less likely to discontinue contraceptive methods [19]. Discussing family planning with a spouse is also a characteristic that is consistently associated with contraceptive use or adoption [30–32]. Women with more education are also more likely to adopt contraception by follow-up.

Given that the modern contraceptive prevalence and method mix differ across these five countries, it is perhaps not surprising that the specific factors associated with adoption also vary across country. Only a few results are consistent across countries (e.g., women with higher parity and who discuss family planning with their partner are more likely to adopt contraception), and there are more differences than similarities. Some characteristics are associated with adoption in only one country: for example, level of education is associated with adoption only in India, and discussing FP with facility personnel is associated with adoption only in Nigeria. Other characteristics have the opposite relationship with adoption across countries: household wealth ranking of five (out of ten) was associated with greater odds of adopting modern contraception in Nigeria (compared to the lowest wealth category) but associated with lower odds in DRC. Women who visited the facility for general health or other reasons have lower odds of adoption on Burkina Faso but higher odds in Nigeria.

Our results have implications for family planning policies in these countries. First, we note that only a small percentage of women reported discussing family planning with facility representatives in all countries. Since our results suggest that these discussions may lead to contraceptive adoption (keeping in mind the caveat above), expanding these discussions of FP may be effective in meeting needs for contraception. Similarly, as found elsewhere [30–32], the importance of discussing FP with a spouse is also notable and may therefore be a worthwhile focus for FP programs. Finally, the connection between facility type and contraceptive adoption has potentially important implications, as it may provide guidance to FP programs for improving service quality and certain facility types.

This research has several notable assets. First, characteristics predicting contraceptive adoption, as opposed to use, have not been extensively studied in developing settings, so we add to a limited body of research. Second, unlike cross-sectional studies, our longitudinal study design allows us to establish the order of events between contraceptive adoption and various characteristics. As a result, we can distinguish the factors that *predict* contraceptive adoption from factors that may be impacted by adoption. In addition, our longitudinal design does not involve retrospective reporting of contraceptive use, which is known to be inaccurate at times. Third, our study includes not only characteristics of the woman, but also of the health facility. It is evident from our analysis that the factors that influence contraceptive use go beyond just individual characteristics; these environmental factors like facility characteristics are important as well, again illustrating the influence of quality of care on contraceptive use. Finally, we include data for five different countries, which vary by contraceptive prevalence and method mix, which enhances the scope of our results.

There are some limitations to our research. We do not have the exact time of adoption and are therefore unable to measure the timing of adoption (and differences between women in earlier compared with later adoption). However, as mentioned above, the measures of time from the contraceptive calendar are often imprecise, which calls into question the accuracy of such analysis. We cannot distinguish if women are adopting contraception for the first time or if they are previous users who discontinued, it might be that first time users are more motivated to adopt contraception than those who discontinued. Finally, although our study includes a range of likely influences on contraceptive adoption, we are missing other important characteristics, such as fertility or pregnancy intentions [33], or duration of time since most recent childbirth [19], and information on where the adopters obtained their contraception by follow-up.

To what extent do our results here apply to broader populations in these countries? We offer several notes on the generalizability of our findings. First, as described in the PMA Agile Cohort Profile [22], although PMA Agile facilities were selected to be representative of all facilities in Agile cities, the clients in PMA Agile facilities are not population-based and are therefore limited in generalizability- which is typical for facility-based studies [19]. Research has found that women who visit clinics have higher levels of education, wealth, and autonomy [34]; therefore, women who visit facilities may be different from those who do not in contraception adoption. In addition, not all women interviewed in the CEI were re-interviewed in the follow-up: follow-up participation rates averaged 70.2% across all sites and countries. There were two primary reasons for not interviewing eligible women in the CEI phone follow-up: either the woman didn’t have or provide a phone number, or the study was unable to reach them for the phone interview. The percentages who didn’t provide a phone number ranged from 1.8% in Kenya to 30.4% in India; and the percentages of women not reached for phone interview ranged from 9.1% in Kenya to 36.8% in India. We examined differences in sociodemographic characteristics between women who were re-interviewed and those who were lost to follow-up (age, parity, wealth, marital status, level of education), and we found that women who were lost to follow-up were significantly younger, had less wealth, were less likely to be married, and had less education (S4 Table).

Despite the importance of measuring women who initiate contraceptive use, the characteristics associated with contraceptive initiation are not well known. Using data from women and family planning service delivery points, we find that both women, partner, and facility characteristics are importantly related to adopting a contraceptive method. The importance of some of the socioeconomic influences on contraceptive use, like age, education, and parity, have been well-established in the literature; but other measures, such as facility characteristics, have been examined less often. A longitudinal approach is also necessary to more accurately examine some measures that could both predict and be affected by contraceptive use, such as discussing family planning with a partner or the staff at a health facility.

## Supporting information

S1 Fig Percentage distribution of modern contraceptive methods adopted at follow-up by baseline nonusers for DRC, India, Kenya, Nigeria, and Burkina Faso. (TIF)

S1 Table Harmonization of facility types across countries, number of clients. (TIF)

S2 Table Logistic regression model of service delivery point characteristics associated with the probability that a client becomes an adopter over a continued nonuser in the Democratic Republic of Congo, India, Kenya, Nigeria, and Burkina Faso. (TIF)

S3 Table Logistic regression model of individual characteristics associated with the probability that a client becomes an adopter over a continued nonuser in the Democratic Republic of Congo, India, Kenya, Nigeria, and Burkina Faso. (TIF)

S4 Table Differences in characteristics between women re-interviewed and those lost to follow-up (LTFU), PMA Agile. (TIF)
