# Supplementary figures and images for: Service delivery point and individual characteristics associated with the adoption of modern contraceptive: A multi-country longitudinal analysis
Source: PLoS One. 2021 Aug 17;16(8):e0254775. doi: 10.1371/journal.pone.0254775 (PMC8370635; doi:10.1371/journal.pone.0254775)

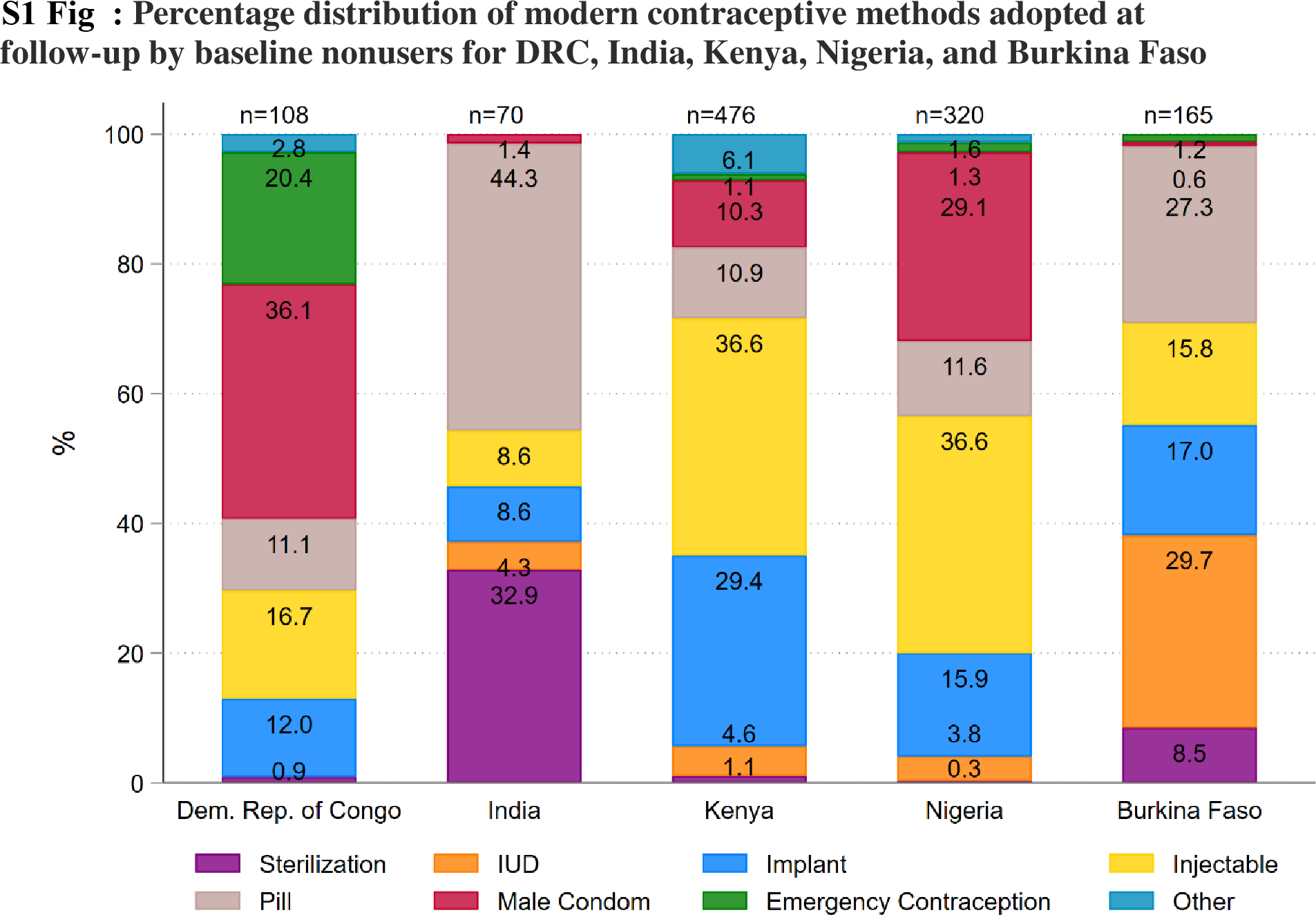

Supplement: S1 Fig — (TIF) [file pone.0254775.s001.tif]

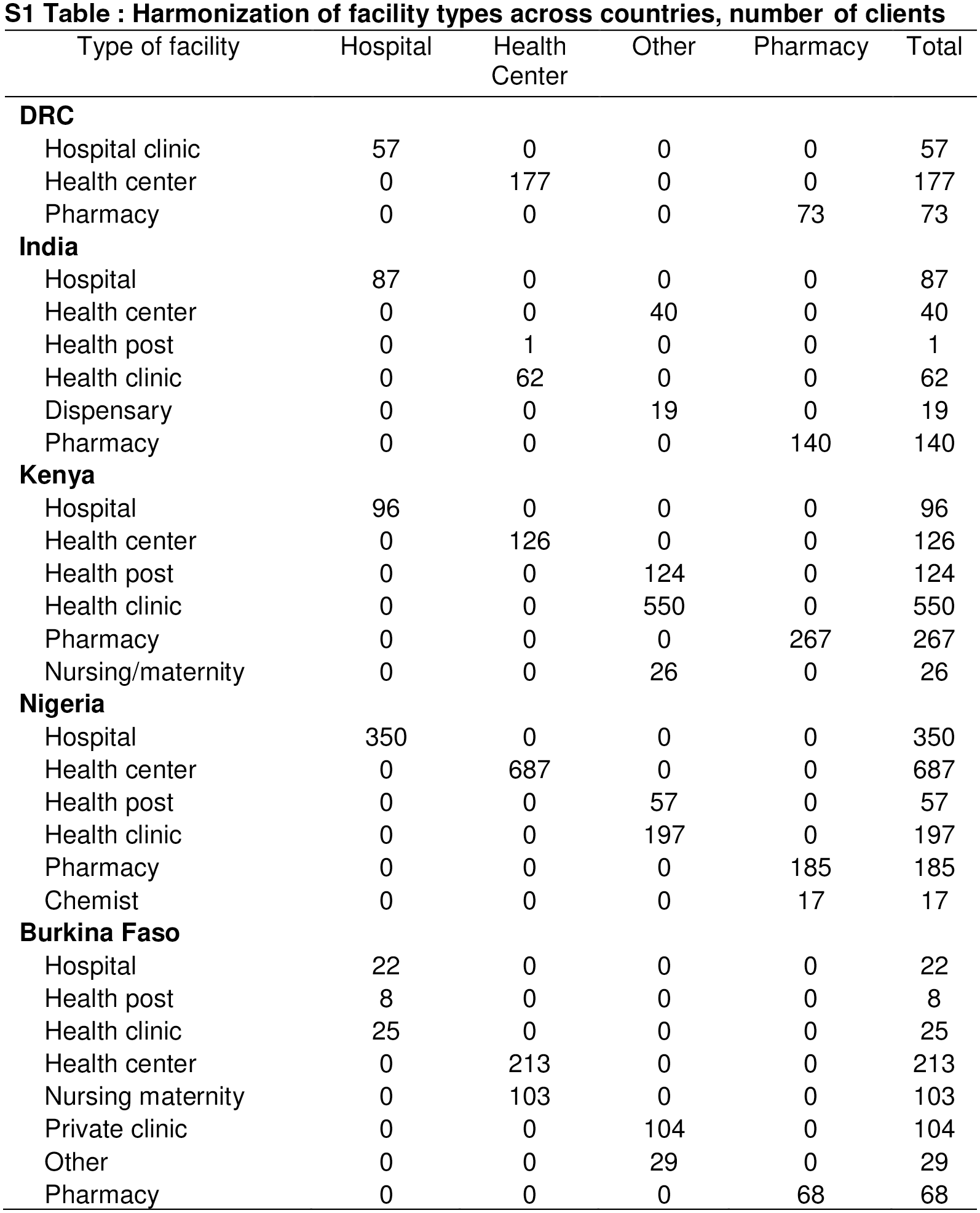

Supplement: S1 Table — (TIF) [file pone.0254775.s002.tif]

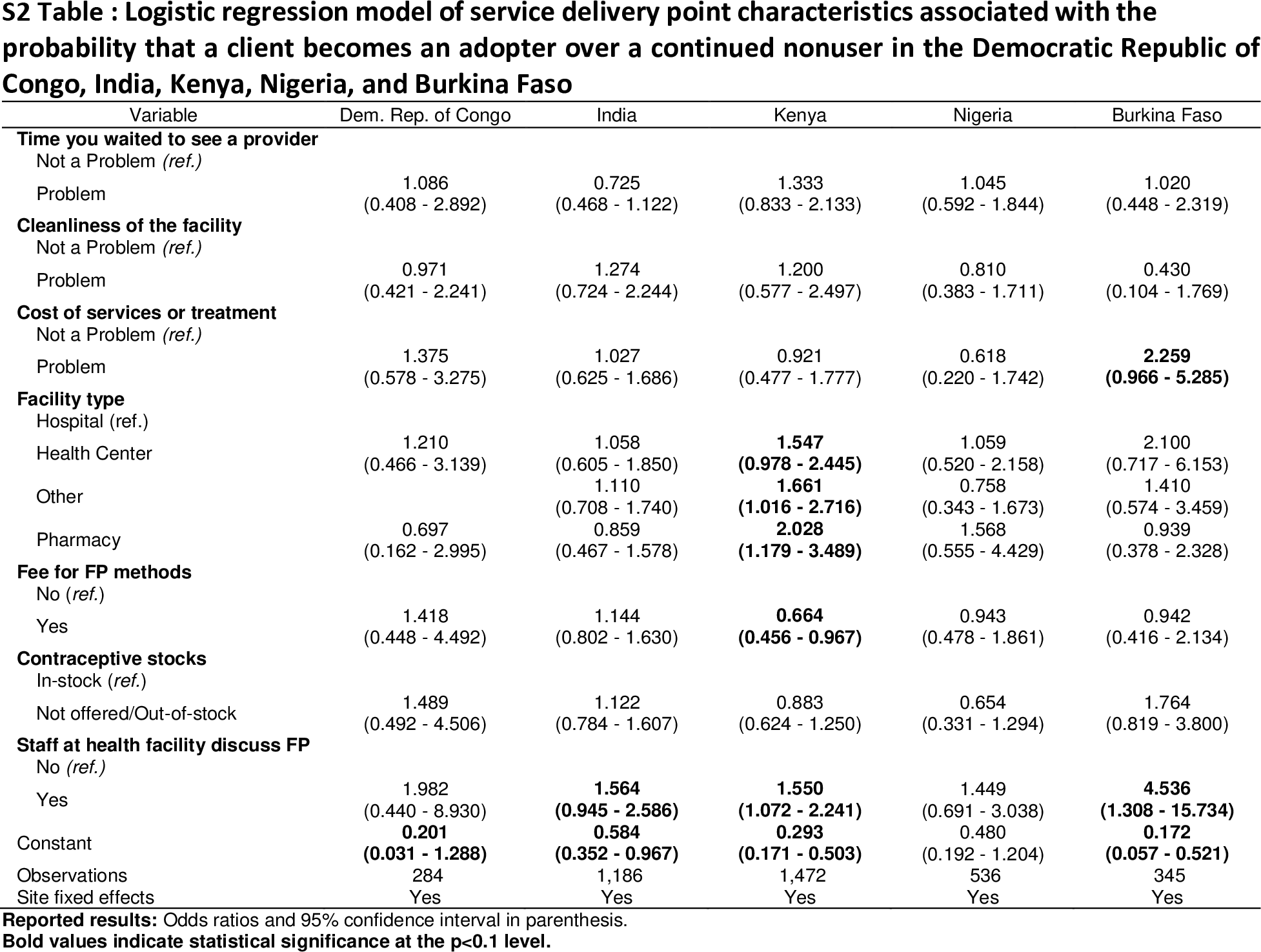

Supplement: S2 Table — (TIF) [file pone.0254775.s003.tif]

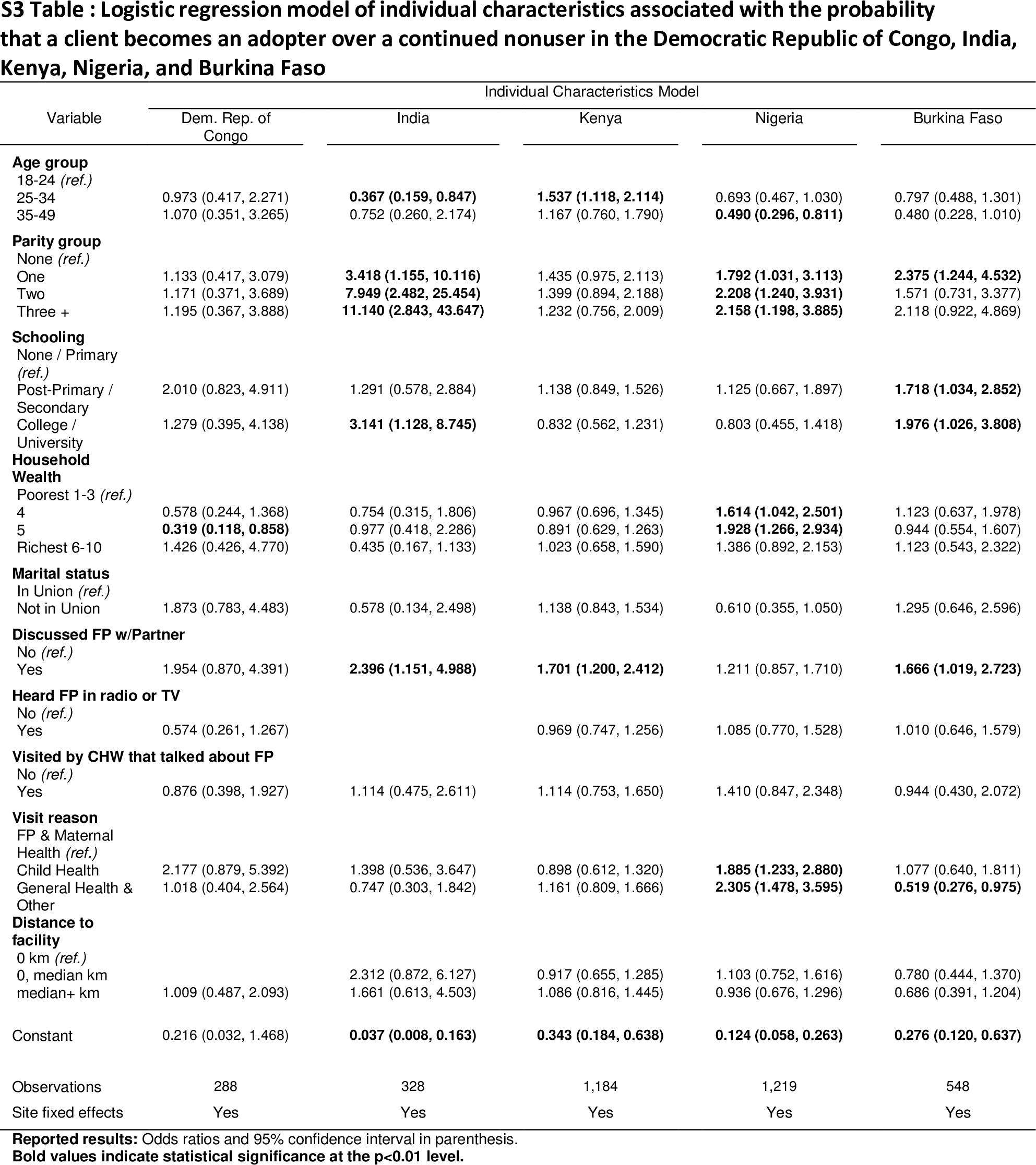

Supplement: S3 Table — (TIF) [file pone.0254775.s004.tif]

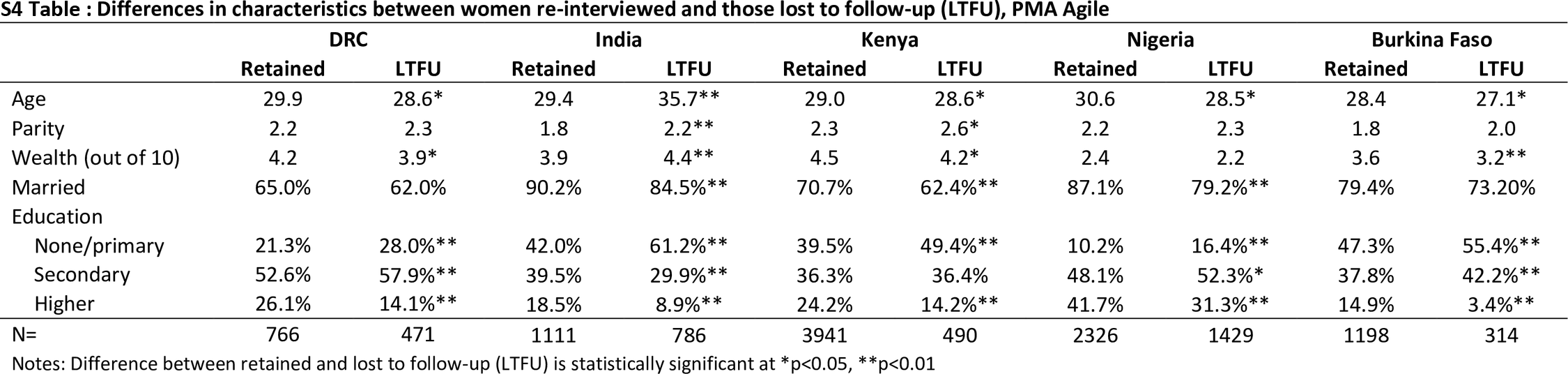

Supplement: S4 Table — (TIF) [file pone.0254775.s005.tif]
